# Recent advances in research on multistage tumorigenesis

**DOI:** 10.1054/bjoc.2000.1309

**Published:** 2000-06-02

**Authors:** A Compagni, G Christofori

**Affiliations:** Research Institute of Molecular Pathology, Dr. Bohr-Gasse 7, Vienna, A-1030, Austria

**Keywords:** cancer, oncogenes, transgenic mice, tumour suppressor genes

## Abstract

Tumour development is a multi-step process during which genetic and epigenetic events determine the transition from a normal to a malignant cellular state. In the past decade, extensive effort has been made not only to define the molecular mechanisms underlying progression to malignancy but also to predict the development of the disease and to identify possible molecular targets for therapy. Common to most tumours, several regulatory circuits are altered during multistage tumour progression, most importantly, the control of proliferation, the balance between cell survival and programmed cell death (apoptosis), the communication with neighbouring cells and the extracellular matrix, the induction of tumour neovascularization (angiogenesis) and, finally, tumour cell migration, invasion and metastatic dissemination. De-regulation of each of these processes represents a rate-limiting step for tumour development and, hence, has to be achieved by tumour cells in a highly selective manner during tumour progression.

In this review we summarize recent advances in cancer research that have provided new insights in the molecular mechanisms underlying the transition between one tumour stage and the next and into their concerted action during tumour progression. Cultured human tumour cell lines as well as transgenic and knock-out mouse models of tumorigenesis have been instrumental in these experimental approaches. © 2000 Cancer Research Campaign


*British Journal of Cancer* (2000) **83**(1), 1–5
© 2000 Cancer Research Campaign
doi: 10.1054/ bjoc.2000.1309, available online at http://www.idealibrary.com on IDEAL®

Millennium mini-review

### Recent advances in research on multistage tumorigenesis

**A Compagni and G Christofori**

Research Institute of Molecular Pathology, Dr. Bohr-Gasse 7, A-1030 Vienna, Austria

**Summary** Tumour development is a multi-step process during which genetic and epigenetic events determine the transition from a normal to a malignant cellular state. In the past decade, extensive effort has been made not only to define the molecular mechanisms underlying progression to malignancy but also to predict the development of the disease and to identify possible molecular targets for therapy. Common to most tumours, several regulatory circuits are altered during multistage tumour progression, most importantly, the control of proliferation, the balance between cell survival and programmed cell death (apoptosis), the communication with neighbouring cells and the extracellular matrix, the induction of tumour neovascularization (angiogenesis) and, finally, tumour cell migration, invasion and metastatic dissemination. De-regulation of each of these processes represents a rate-limiting step for tumour development and, hence, has to be achieved by tumour cells in a highly selective manner during tumour progression.

In this review we summarize recent advances in cancer research that have provided new insights in the molecular mechanisms underlying the transition between one tumour stage and the next and into their concerted action during tumour progression. Cultured human tumour cell lines as well as transgenic and knock-out mouse models of tumorigenesis have been instrumental in these experimental approaches. © 2000 Cancer Research Campaign

**Keywords:** cancer; oncogenes; transgenic mice; tumour suppressor genes

### PROLIFERATION CONTROL

Cancer cells bear an indefinite proliferative capacity being able to elude the commitment to terminal differentiation and quiescence that normally regulate tissue homeostasis in the organism. To achieve this property, tumour cells become independent from external growth stimuli. For example, glioblastoma and sarcoma cells produce their own growth factors, (PDGF) and transforming growth factor α (TGF-α) respectively, that are normally produced by stromal cells (Fedi et al, 1997). Similarly, melanoma cells produce high levels of fibroblast growth factor 2 (FGF-2) and are dependent on this growth factor for proliferation (Becker et al, 1989). On the other hand, the signalling pathways that are involved in the transduction of mitogenic stimuli are often constitutively activated in tumour cells, either by overexpression or by mutation of signal transduction molecules. Examples are the up-regulated expression of growth-factor receptors, such as epidermal growth factor (EGF) receptor or HER-2/neu (Yarden and Ullrich, 1988). Moreover, tyrosine-kinase receptors, such as EGF receptor, are frequently found mutated or truncated in a way that they are constitutively active independently from ligand binding (Fedi et al, 1997). Finally, 25% of human tumours present activating mutations in ras, resulting in persistent signal transduction via the MAP kinase pathway, the PI-3 kinase pathway, and possibly other downstream effector pathways. Nonetheless, the transforming potential of ras often depends on the cell type and possibly on its expression levels. For example, in primary cells introduction of an activated form of ras or constitutive activation of the Raf/MAP kinase pathway induces cell senescence instead of proliferation (Serrano et al, 1997; Lin et al, 1998; Zhu et al, 1998). In contrast, in tumour-prone INK4a-deficient mice expression of an activated Ha-ras is sufficient to induce melanoma formation (Chin et al, 1999*b*). Notably, the maintenance of the transformed state is dependent on ras activity, since tumour growth is reversed when expression of the oncogene is abolished. Similarly to ras, induction of c-myc expression in skin or T lymphocytes leads to neoplasia in a reversible fashion (Felsher and Bishop, 1999; Pelengaris et al, 1999). These experiments indicate that the sole activation of an oncogene can instruct cells to proliferate and prevent them from differention, growth arrest, or apoptosis. Nonetheless, tumour cells seem to retain the capability of returning to normal regulatory responses, when the oncogene is inactivated. In contrast to these results, SV40 T antigen-mediated transformation of pancreatic β-cells could not be reversed after a certain time point (Ewald et al, 1996). These experiments solicit questions about the number and nature of additional genetic or epigenetic changes that render tumour cells irreversibly malignant.

Most signal transduction pathways directly or indirectly affect cell cycle regulation, and tumour cells achieve the capability to indefinitely proliferate also by directly de-regulating cell cycle control. Such events mainly involve functional changes in the tumour suppressor retinoblastoma (pRb) and components that are affected by its function. In many cancer types and experimental tumours, pRb itself is disabled, for example by mutation or deletion of the gene or by interaction with viral oncoproteins (SV40 T antigen, human papilloma virus E7, adenovirus E1A). Alternatively, the function of molecules that act in the pRb pathway are frequently impaired. For example, the gene for the cyclin dependent kinase (cdk)-inhibitor p16 is often mutated or

*Received 28 February 2000*
*Accepted 3 March 2000*

*Correspondence to:* G Christofori lost, while cyclin D1 and cdk4 are overexpressed (Chin et al, 1998). These changes allow cancer cells to enter the cell cycle, to maintain its progression and to escape growth-arrest signals.

A novel pathway for the onset of tumour cell proliferation has recently been discovered by studying the function of the tumour suppressor adenomatous polyposis coli (APC), mainly in colon cancer and melanoma development. APC is mutated in a hereditary form of colon cancer, familial adenomatous polyposis (FAP), and in sporadic forms of colon cancer which invariably leads to hyperproliferation of colon crypt cells and to the formation of polyps (Kinzler and Vogelstein, 1996; Polakis, 1997). It has been demonstrated that APC plays a central role in Wnt signalling. Among several other proteins APC binds β-catenin and exposes it to glycogen synthase kinase 3β (GSK3β) which phosphorylates β-catenin and earmarks it for ubiquitination and degradation by the proteasome pathway. In case APC function is lost, β-catenin accumulates in the cytoplasm. A subset of β-catenin is imported into the nucleus where it binds members of the LEF-1/TCF transcription factor family and activates transcription of target genes (Bullions and Levine, 1998; Eastman and Grosschedl, 1999), including c-myc and cyclin D1 (He et al, 1998; Shtutman et al, 1999; Tetsu and McCormick, 1999). These results revealed the long awaited molecular connection between the loss of APC function and tumour cell proliferation. The same outcome, i.e. up-regulation of cyclin D1 and c-myc and thus the induction of proliferation, is achieved in melanoma cells by mutations in β-catenin that render it resistant to phosphorylation by GSK3β. This results in constitutively up-regulated β-catenin/TCF-mediated transcriptional activity (Korinek et al, 1997; Morin et al, 1997; Rubinfeld et al, 1997).

### SURVIVAL SIGNALS

The activation of oncogenes, such as ras and c-myc, not only induces proliferation and transformation but also an apoptotic signal mediated by the tumour suppressor p53. The connection between proliferation and the induction of apoptosis by p53 has been recently elucidated (Sherr, 1998). Up-regulated expression of c-myc induces the expression of p19/ARF which subsequently binds and inactivates mdm-2, a proto-oncogene that mediates the degradation of p53 (Zindy et al, 1998). Stabilization and accumulation of p53 results in transcriptional activation of several p53 target genes, including the cell cycle inhibitor p21, the apoptosis inducer bax and several other genes that are known to directly or indirectly induce apoptosis. Moreover, tumour cell apoptosis is also frequently induced by up-regulated expression of Fas ligand which in turn binds to Fas receptor expressed on tumour cells (Hueber et al, 1997). The apoptotic response to unscheduled proliferation possibly constitutes a general defence mechanism to prevent transformation (Sherr, 1998). This notion has not only given further justification to the high percentage of p53 mutations found in human cancer, but also has emphasized the importance of escaping apoptosis for tumour growth. Tumour cells frequently up-regulate the expression of anti-apototic genes, such as bcl-2, to counteract induction and execution of apoptosis (Jaattela, 1999). Alternatively, many tumour types acquire the expression of growth factors, such as IGFs and PDGFs, which act as survival signals. Growth factor-mediated survival has been first demonstrated in vitro by transformation of fibroblasts; c-myc-expressing fibroblasts rapidly die upon serum-starvation and are rescued from apoptosis by a number of growth factors, including IGFs, PDGF and FGFs (Harrington et al, 1994). In vivo, the role of IGF-II as a survival signal has been demonstrated in a transgenic mouse model of β-cell carcinogenesis (Christofori et al, 1994). IGFs mediate survival by binding their cognate receptor, the IGF-1 receptor, and the subsequent activation of PI-3 kinase and PKB/Akt (Downward, 1998; Baserga, 1999). Curiously, PTEN, a major tumour suppressor gene that is mutated in a large fraction of human cancer types, is able to impair survival factor function (Ali et al, 1999). PTEN, as a bifunctional phosphatase, is able to dephosphorylate both phosphoserines on proteins and phospatidylinositol phosphates, thereby directly counteracting PI-3 kinase activity and thus the anti-apoptotic function of PKB/Akt (Stambolic et al, 1998).

### SENESCENCE

Growth inhibition and apoptosis are not the only mechanisms that counteract tumour progression. Cells have also developed a device, based on their telomere length, to count the number of cell doublings. Normal cells, after a limited number of divisions (60–70 for human cells) enter crisis and, as a result, arrest in cell cycle and enter a senescent state. Telomerase, the enzyme that is responsible to maintain proper telomere length, is not active in normal somatic cells, however, its activity is found to be up-regulated in approximately 80% of cancer cells (Holt and Shay, 1999), prolonging their life span and allowing them to circumvent crisis and senescence (Bodnar et al, 1998). This concept is best illustrated by experiments in which tumour-prone INK4a-deficient mice were crossed with telomerase-deficient mice (mTR−/−). In the absence of telomerase activity, tumour incidence was greatly reduced (Greenberg et al, 1999), indicating that the gain of telomerase activity indeed favours tumour progression. On the other hand, two observations contradict this conclusion and complicate the evaluation of telomerase as a target for therapeutic intervention. First, telomerase-deficient mice, when bred up to the sixth generation, exhibit a significant tumour predisposition (Rudolph et al, 1999). Secondly, embryonic fibroblasts deficient for both p53 and telomerase have a higher transformation rate than p53-deficient cells (Chin et al, 1999*a*). In both cases, the lack of telomerase activity correlates with a high rate of chromosomal damage and overall genomic instability. Telomere erosion is now known to be recognized by DNA damage-sensing systems resulting in the induction of p53 (Karlseder et al, 1999; Zhang et al, 1999). Therefore, it can be speculated, that in the absence of p53, cells do not arrest upon DNA damage and continue to accumulate chromosomal abnormalities, thus promoting tumour progression.

### Co-operative effects

Several groups have attempted to define the minimum number of alterations that lead to the transformation of human cells. The results are contradictory. Weinberg and co-workers succeeded in transforming normal human epithelial cells and fibroblasts with a combination of SV40 T antigen (to inactivate the two tumour suppressors p53 and pRb), human telomerase (hTERT) and activated ras (Hahn et al, 1999). In contrast, a previous report has shown that the combination of hTERT, human papillomavirus E6/E7 oncoproteins (to inactivate p53 and pRb), and activated ras is not sufficient to induce transformation of human primary cells (Morales et al, 1999). The reason for this discrepancy is not clear,

 © 2000 Cancer Research Campaign however, it is possible that the sequence of events may be critical for full transformation.

### Tumour angiogenesis

Already in the 1960s, Folkman and co-workers suggested that tumour growth critically depends on the formation of new blood vessels (angiogenesis) (Folkman, 1990). Since then, it has been demonstrated that the angiogenic process is the result of a fine balance between inducers and inhibitors (Folkman, 1995; Hanahan and Folkman, 1996). Hence, induction of tumour angiogenesis can be caused by the up-regulation of angiogenic factors or by the loss of inhibitors. Among the angiogenic factors, vascular endothelial growth factor (VEGF-A) is frequently up-regulated during tumorigenesis, and its involvement in tumour progression has been repeatedly proven (Ferrara, 1999). Notably, expression of VEGF-A is high in hypoxic tumour areas and it is promoted by the hypoxia inducible factor 1α (HIF-1α), a transcription factor that regulates many other hypoxia-induced genes (Semenza, 1999).

Many naturally occurring and experimentally designed angiogenesis inhibitors have been reported (Bouck et al, 1996). Among the endogenous angiostatic factors, thrombospondin-1 (TSP-1) has been identified as a direct transcriptional target of the tumour suppressor p53 (Dameron et al, 1994). By binding to its receptor CD36 on endothelial cells, TSP1 activates at least two distinct signal transduction pathways that lead to endothelial cell apoptosis (Jimenez et al, 2000). By virtue of its protease activity TSP-1 is also able to activate the latent form of TGF-β, known for its cytostatic activity on endothelial cells (Crawford et al, 1998). Loss of p53, therefore, also contributes to the onset of tumour angiogenesis. A similar connection between p53 function and tumour angiogenesis has been recently demonstrated. It has been shown that p53 targets HIF-1α to degradation via mdm-2. Conversely, upon loss of p53, HIF-1α is no more degraded, thus supporting tumour angiogenesis and with it tumour growth (Ravi et al, 2000). Of course, inhibition of tumour angiogenesis is a major therapeutic opportunity, and many of the compounds that are able to interfere with tumour angiogenesis are already in clinical trials (Ferrara and Alitalo, 1999).

### Invasion and metastasis

The final stages of tumour progression are characterized by the invasion of tumour cells into their surrounding tissue and the dissemination of tumour cells to form metastases in distant organs. The molecular mechanisms underlying the cellular changes during these events are still not fully understood, however, there is a general consensus that cell-to-cell and cell-to-substrate interactions have to be profoundly altered during these final stages.

E-cadherin is the prototypic cell–cell adhesion molecule involved in the formation of adherens junctions in epithelia. It is functionally inactivated in virtually all human epithelial cancers (carcinomas) by gene deletion or mutation, promoter silencing and proteolytic cleavage of the protein (Christofori and Semb, 1999). Moreover, germline mutations in the E-cadherin gene predispose patients to diffuse and poorly differentiated gastric cancer (Guilford et al, 1998). Experiments with a transgenic mouse model for pancreatic β-cell carcinogenesis have demonstrated that the loss of E-cadherin is causally involved in the transition from a benign to a malignant tumour stage (Perl et al, 1998). However, it remains elusive how the loss of E-cadherin-mediated cell–cell adhesion induces active tumour cell invasion and metastasis. Since the functional E-cadherin cell adhesion complex, among other proteins, also contains β-catenin, it is conceivable that upon loss of E-cadherin function β-catenin is released, translocates to the nucleus and together with LEF-1/TCF transcription factors induces specific transcriptional programmes (Christofori and Semb, 1999).

Mainly based on in vitro experiments in which forced expression of proteases or inhibition of their activity altered the invasiveness of tumour cells, proteases have been assumed to play a functional role only in late stage tumour progression. Unexpectedly, this scenario has changed. For example, the metalloprotease matrilysin (MMP-7) is already expressed in the early stages of human colorectal cancer (Fingleton et al, 1999) and has been shown to be a direct target of the transcriptional complex β-catenin/TCF (Crawford et al, 1999). Notably, intestinal tumorigenesis in a mouse strain carrying a mutated allele of the APC tumour suppressor gene (Min mouse) is dramatically repressed in the absence of matrilysin (Wilson et al, 1997). Surprisingly, expression of stromelysin-1 (MMP-3) in the mammary gland of transgenic mice appears to be sufficient to induce full-blown carcinogenesis, linking protease activity with tumour initiation and possibly proliferation control (Sternlicht et al, 1999). On the other hand, gelatinase A (MMP-2) and the uPA/uPAR system have been shown to play an important role in tumour angiogenesis (Brooks et al, 1998; Rabbani, 1998; Coussens et al, 1999). Thus, proteases act at multiple stages during tumour development and therefore offer valuable targets for therapeutic intervention.

### Mutator phenotypes

As discussed above, most of the events involved in the progression to cancer represent genetic (mutational) changes that directly affect proliferation, survival and transformation of tumour cells. However, changes in basic regulatory mechanisms, such as DNA repair or DNA methylation, also affect neoplastic transformation by accelerating tumour progression.

In patients with hereditary non-polyposis colorectal cancer (HNPCC), most tumours show microsatellite instability (MIN). Microsatellites are highly repetitive DNA sequences that are prone to accumulate errors (mismatches) during DNA replication. In HNPCC patients, the failure to repair these mismatches is due to inactivating mutations in genes involved in mismatch repair, such as MLH1 and MSH2 (Peltomaki and de la Chapelle, 1997). Defects in the mismatch repair system can shift the spontaneous mutation rate from $10^{-6}$ to $10^{-3}$–$10^{-2}$, resulting in a so-called mutator phenotype characterized by an increased frequency of mutations in tumour suppressor genes and oncogenes. Indeed, microsatellites have been found within the coding regions of genes that are known to play important roles in cellular transformation, including APC, TGF-β receptor II, IGF-2 receptor, TCF-4, bax and the mismatch repair genes themselves (Duval et al, 1999; Schwartz et al, 1999). A failure to repair replication errors within these microsatellites invariably leads to a loss of the tumour suppressing functions of these genes.

In contrast to the genetic instability caused by DNA repair defects (MIN), chromosomal instability (CIN) is characterized by chromosomal rearrangements, including large deletions, fusions and translocations (Lengauer et al, 1998). CIN is easily recognized in tumour cells by their aneuploid DNA content. On a molecular level, a first report indicates a correlation between aneuploidy and

© 2000 Cancer Research Campaign mutations in molecules responsible for chromosome separation during mitosis (Cahill et al, 1998), however, a causal role of these mutations in tumour initiation is still unclear. Evidence for an involvement of chromosomal instability in cancer comes from studies on DNA repair in response to double-strand DNA breaks. Breaks in the DNA double helix, caused for example by γ-irradiation, usually induce activation of p53 via the kinases ATM/ATR or alternatively of the p53 family member p73 via ATM and c-abl, resulting in growth arrest or apoptosis (Dasika et al, 1999). Several molecules involved in this pathway, such as ATM, or proteins that may be directly involved in the repair mechanisms, such as BRCA-1 and -2, are known tumour suppressor genes that are frequently lost in several types of cancer. Knock-out mice deficient for any of these genes develop tumours that exhibit genomic instability, including aneuploidy and chromosomal rearrangements (Dasika et al, 1999). DNA breaks are not only induced by defects in sister chromatid separation during mitosis or by DNA damaging agents. There are, in fact, sites in the genome that show high predisposition to DNA breaks (fragile sites). Coincidentally, another tumour suppressor gene, FHIT, is localized within one of these fragile sites, and errors in repairing DNA breaks are likely the cause of deletions observed in the FHIT gene (Huebner et al, 1998).

Besides mutation or deletion, tumour suppressor genes can also be disabled by silencing their promoters. Recent studies have demonstrated that in many different tumour cell types CpG islands in the promoter region of many tumour suppressor genes are hypermethylated, thereby preventing their expression (Toyota and Issa, 1999). The frequency of this CpG island methylator phenotype (CIMP) accounts for most of the tumour cases in which mutations or chromosomal aberrations affecting tumour suppressor genes could not be detected. Tumour suppressor genes that are inactivated by CIMP include the von Hippel Lindau gene (VHL), the cell cycle inhibitor p16/INK4a, and the cell adhesion molecule E-cadherin (Herman, 1999). Curiously, the mismatch repair gene MLH1 has also been found inactivated by hypermethylation, suggesting that CIMP may lead to the MIN mutator phenotype (Herman, 1999). Hypermethylation does not appear to be a random process, since some promoters seem to be predisposed (Herman, 1999). However, the molecular mechanisms by which promoter hypermethylation is achieved and how its specificity is acquired are not known. There is only indirect evidence that alterations in DNA methylase (Mtase) activity could account for CIMP. Mtase activity is frequently found to be increased in various human malignancies, including colon cancer, haematopoietic cancers and lung cancer (Toyota and Issa, 1999). Moreover, inhibition of Mtase activity in mice, that by mutation of the APC tumour suppressor gene are predisposed to colon cancer (Min mice), markedly reduced tumour incidence (Laird et al, 1995).

The general role of mutator phenotypes in carcinogenesis, in particular in cancer types other than colon cancer, will be an interesting issue for the future.

### CONCLUSION

Although still in its early days, molecular cancer research has accomplished extraordinary progress in the understanding of the molecular mechanisms underlying tumour progression. In addition to tumour cell lines in culture, transgenic mouse models of tumorigenesis have been instrumental in deciphering the molecular players causally involved in the transition from one tumour stage to the next. Moreover, basic research has begun to integrate knowledge about the role of exogenous stimuli, including environmental, occupational and dietary influences, into molecular pathways involved in multistage tumorigenesis. Thus, it is exciting times not only for the full understanding of how tumours develop but also for the discovery of novel appropriate targets for therapeutic intervention.

### ACKNOWLEDGEMENTS

The work in the laboratory of the authors was supported by Boehringer Ingelheim, Germany, and by the Austrian Industrial Research Promotion Fund.

### REFERENCES

Ali IU, Schriml LM and Dean M (1999) Mutational spectra of PTEN/MMAC1 gene: a tumor suppressor with lipid phosphatase activity. *J Natl Cancer Inst* **91**: 1922–1932

Baserga R (1999) The IGF-I receptor in cancer research. *Exp Cell Res* **253**: 1–6

Becker D, Meier CB and Herlyn M (1989) Proliferation of human malignant melanomas is inhibited by antisense oligodeoxynucleotides targeted against basic fibroblast growth factor. *Embo J* **8**: 3685–3691

Bodnar AG, Ouellette M, Frolkis M, Holt SE, Chiu CP, Morin GB, Harley CB, Shay JW, Lichtsteiner S and Wright WE (1998) Extension of life-span by introduction of telomerase into normal human cells. *Science* **279**: 349–352

Bouck N, Stellmach V and Hsu SC (1996) How tumors become angiogenic. *Adv Cancer Res* **69**: 135–174

Brooks PC, Silletti S, von Schalscha TL, Friedlander M and Cheresh DA (1998) Disruption of angiogenesis by PEX, a noncatalytic metalloproteinase fragment with integrin binding activity. *Cell* **92**: 391–400

Bullions LC and Levine AJ (1998) The role of beta-catenin in cell adhesion, signal transduction, and cancer. *Curr Opin Oncol* **10**: 81–87

Cahill DP, Lengauer C, Yu J, Riggins GJ, Wilson JK, Markowitz SD, Kinzler KW and Vogelstein B (1998) Mutations of mitotic checkpoint genes in human cancers. *Nature* **392**: 300–303

Chin L, Pomerantz J and DePinho RA (1998) The INK4a/ARF tumor suppressor: one gene – two products – two pathways. *Trends Biochem Sci* **23**: 291–296

Chin L, Artandi SE, Shen Q, Tam A, Lee SL, Gottlieb GJ, Greider CW and DePinho RA (1999*a*). p53 deficiency rescues the adverse effects of telomere loss and cooperates with telomere dysfunction to accelerate carcinogenesis. *Cell* **97**: 527–538

Chin L, Tam A, Pomerantz J, Wong M, Holash J, Bardeesy N, Shen Q, O'Hagan R, Pantginis J, Zhou H, Horner JW, 2nd, Cordon-Cardo C, Yancopoulos GD and DePinho RA (1999*b*). Essential role for oncogenic Ras in tumour maintenance. *Nature* **400**: 468–472

Christofori G and Semb H (1999) The role of the cell-adhesion molecule E-cadherin as a tumour-suppressor gene. *Trends Biochem Sci* **24**: 73–76

Christofori G, Naik P and Hanahan D (1994) A second signal supplied by insulin-like growth factor II in oncogene-induced tumorigenesis. *Nature* **369**: 414–418

Coussens LM, Raymond WW, Bergers G, Laig-Webster M, Behrendtsen O, Werb Z, Caughey GH and Hanahan D (1999) Inflammatory mast cells up-regulate angiogenesis during squamous epithelial carcinogenesis. *Genes Dev* **13**: 1382–1397

Crawford HC, Fingleton BM, Rudolph-Owen LA, Goss KJ, Rubinfeld B, Polakis P and Matrisian LM (1999) The metalloproteinase matrilysin is a target of beta-catenin transactivation in intestinal tumors. *Oncogene* **18**: 2883–2891

Crawford SE, Stellmach V, Murphy-Ullrich JE, Ribeiro SM, Lawler J, Hynes RO, Boivin GP and Bouck N (1998) Thrombospondin-1 is a major activator of TGF-betal in vivo. *Cell* **93**: 1159–1170

Dameron KM, Volpert OV, Tainsky MA and Bouck N (1994) Control of angiogenesis in fibroblasts by p53 regulation of thrombospondin-1. *Science* **265**: 1582–1584

Dasika GK, Lin SC, Zhao S, Sung P, Tomkinson A and Lee EY (1999) DNA damage-induced cell cycle checkpoints and DNA strand break repair in development and tumorigenesis. *Oncogene* **18**: 7883–7899

Downward J. (1998) Mechanisms and consequences of activation of protein kinase B/Akt. *Curr Opin Cell Biol* **10**: 262–267

Duval A, Iacopetta B, Ranzani GN, Lothe RA, Thomas G and Hamelin R. (1999) Variable mutation frequencies in coding repeats of TCF-4 and other target

© 2000 Cancer Research Campaign genes in colon, gastric and endometrial carcinoma showing microsatellite instability. *Oncogene* **18**: 6806–6809

Eastman Q and Grosschedl R (1999) Regulation of LEF-1/TCF transcription factors by Wnt and other signals. *Curr Opin Cell Biol* **11**: 233–240

Ewald D, Li M, Efrat S, Auer G, Wall RJ, Furth PA and Hennighausen L (1996) Time-sensitive reversal of hyperplasia in transgenic mice expressing SV40 T antigen. *Science* **273**: 1384–1386

Fedi P, Tronick SR and Aaronson SA (1997) Growth factors. In: *Cancer Medicine*, Holland JF, Bast RC, Morton DL, Frei E, Kufe DW and Weichselbaum RR (eds) pp 41–64. Williams and Wilkins: Baltimore, MD

Felsher DW and Bishop JM (1999) Reversible tumorigenesis by MYC in hematopoietic lineages. *Mol Cell* **4**: 199–207

Ferrara N (1999) Molecular and biological properties of vascular endothelial growth factor. *J Mol Med* **77**: 527–543

Ferrara N and Alitalo K (1999) Clinical applications of angiogenic growth factors and their inhibitors. *Nat Med* **5**: 1359–1364

Fingleton BM, Heppner Goss KJ, Crawford HC and Matrisian LM (1999) Matrilysin in early stage intestinal tumorigenesis. *Apmis* **107**: 102–110

Folkman J (1990) What is the evidence that tumors are angiogenesis dependent? *J Natl Cancer Inst* **82**: 4–6

Folkman J (1995) Angiogenesis in cancer, vascular, rheumatoid and other disease. *Nat Med* **1**: 27–31

Greenberg RA, Chin L, Femino A, Lee KH, Gottlieb GJ, Singer RH, Greider CW and DePinho RA (1999) Short dysfunctional telomeres impair tumorigenesis in the INK4a(delta2/3) cancer-prone mouse. *Cell* **97**: 515–525

Guilford P, Hopkins J, Harraway J, McLeod M, McLeod N, Harawira P, Taite H, Scoular R, Miller A and Reeve AE (1998) E-cadherin germline mutations in familial gastric cancer. *Nature* **392**: 402–405

Hahn WC, Counter CM, Lundberg AS, Beijersbergen RL, Brooks MW and Weinberg RA (1999) Creation of human tumour cells with defined genetic elements. *Nature* **400**: 464–468

Hanahan D and Folkman J (1996) Patterns and emerging mechanisms of the angiogenic switch during tumorigenesis. *Cell* **86**: 353–364

Harrington EA, Bennett MR, Fanidi A and Evan GI (1994) c-Myc-induced apoptosis in fibroblasts is inhibited by specific cytokines. *Embo J* **13**: 3286–3295

He TC, Sparks AB, Rago C, Hermeking H, Zawel L, da Costa LT, Morin PJ, Vogelstein B and Kinzler KW (1998) Identification of c-MYC as a target of the APC pathway. *Science* **281**: 1509–1512

Herman JG (1999) Hypermethylation of tumor suppressor genes in cancer. *Semin Cancer Biol* **9**: 359–367

Holt SE and Shay JW (1999) Role of telomerase in cellular proliferation and cancer. *J Cell Physiol* **180**: 10–18

Hueber AO, Zornig M, Lyon D, Suda T, Nagata S and Evan GI (1997) Requirement for the CD95 receptor-ligand pathway in c-Myc-induced apoptosis. *Science* **278**: 1305–1309

Huebner K, Garrison PN, Barnes LD and Croce CM (1998) The role of the FHIT/FRA3B locus in cancer. *Annu Rev Genet* **32**: 7–31

Jaattela M (1999) Escaping cell death: survival proteins in cancer. *Exp Cell Res* **248**: 30–43

Jimenez B, Volpert OV, Crawford SE, Febbraio M, Silverstein RL and Bouck N (2000) Signals leading to apoptosis-dependent inhibition of neovascularization by thrombospondin-1. *Nat Med* **6**: 41–418

Karlseder J, Broccoli D, Dai Y, Hardy S and de Lange T (1999) p53- and ATM-dependent apoptosis induced by telomeres lacking TRF2. *Science* **283**: 1321–1325

Kinzler KW and Vogelstein B (1996) Lessons from hereditary colorectal cancer. *Cell* **87**: 159–170

Korinek V, Barker N, Morin PJ, van Wichen D, de Weger R, Kinzler KW, Vogelstein B and Clevers H (1997) Constitutive transcriptional activation by a beta-catenin-Tcf complex in APC−/− colon carcinoma. *Science* **275**: 1784–1787

Laird PW, Jackson-Grusby L, Fazeli A, Dickinson SL, Jung WE, Li E, Weinberg RA and Jaenisch R (1995) Suppression of intestinal neoplasia by DNA hypomethylation. *Cell* **81**: 197–205

Lengauer C, Kinzler KW and Vogelstein B (1998) Genetic instabilities in human cancers. *Nature* **396**: 643–649

Lin AW, Barradas M, Stone JC, van Aelst L, Serrano M and Lowe SW (1998) Premature senescence involving p53 and p16 is activated in response to constitutive MEK/MAPK mitogenic signaling. *Genes Dev* **12**: 3008–3019

Morales CP, Holt SE, Ouellette M, Kaur KJ, Yan Y, Wilson KS, White MA, Wright WE and Shay JW (1999) Absence of cancer-associated changes in human fibroblasts immortalized with telomerase. *Nat Genet* **21**: 115–118

Morin PJ, Sparks AB, Korinek V, Barker N, Clevers H, Vogelstein B and Kinzler KW (1997) Activation of beta-catenin-Tcf signaling in colon cancer by mutations in beta-catenin or APC. *Science* **275**: 1787–1790

Pelengaris S, Littlewood T, Khan M, Elia G and Evan G (1999) Reversible activation of c-Myc in skin: induction of a complex neoplastic phenotype by a single oncogenic lesion. *Mol Cell* **3**: 565–577

Peltomaki P and de la Chapelle A (1997) Mutations predisposing to hereditary nonpolyposis colorectal cancer. *Adv Cancer Res* **71**: 93–119

Perl AK, Wilgenbus P, Dahl U, Semb H and Christofori G (1998) A causal role for E-cadherin in the transition from adenoma to carcinoma. *Nature* **392**: 190–193

Polakis P (1997) The adenomatous polyposis coli (APC) tumor suppressor. *Biochim Biophys Acta* **1332**: F127–147

Rabbani SA (1998) Metalloproteases and urokinase in angiogenesis and tumor progression. *In Vivo* **12**: 135–142

Ravi R, Mookerjee B, Bhujwalla ZM, Sutter CH, Artemov D, Zeng Q, Dillehay LE, Madan A, Semenza GL and Bedi A (2000) Regulation of tumor angiogenesis by p53-induced degradation of hypoxia-inducible factor 1alpha. *Genes Dev* **14**: 34–44

Rubinfeld B, Robbins P, El-Gamil M, Albert I, Porfiri E and Polakis P (1997) Stabilization of beta-catenin by genetic defects in melanoma cell lines. *Science* **275**: 1790–1792

Rudolph KL, Chang S, Lee HW, Blasco M, Gottlieb GJ, Greider C and DePinho RA (1999) Longevity, stress response, and cancer in aging telomerase-deficient mice. *Cell* **96**: 701–712

Schwartz S Jr, Yamamoto H, Navarro M, Maestro M, Reventos J and Perucho M (1999) Frameshift mutations at mononucleotide repeats in caspase-5 and other target genes in endometrial and gastrointestinal cancer of the microsatellite mutator phenotype. *Cancer Res* **59**: 2995–3002

Semenza GL (1999) Regulation of mammalian $O_2$ homeostasis by hypoxia-inducible factor 1. *Annu Rev Cell Dev Biol* **15**: 551–578

Serrano M, Lin AW, McCurrach ME, Beach D and Lowe SW (1997) Oncogenic ras provokes premature cell senescence associated with accumulation of p53 and p161NK4a. *Cell* **88**: 593–602

Sherr CJ (1998) Tumor surveillance via the ARF-p53 pathway. *Genes Dev* **12**: 2984–2991

Shtutman M, Zhurinsky J, Simcha I, Albanese C, D'Amico M, Pestell R and Ben-Ze'ev A (1999) The cyclin D1 gene is a target of the beta-catenin/LEF-1 pathway. *Proc Natl Acad Sci USA* **96**: 5522–5527

Stambolic V, Suzuki A, de la Pompa JL, Brothers GM, Mirtsos C, Sasaki T, Ruland J, Penninger JM, Siderovski DP and Mak TW (1998) Negative regulation of PKB/Akt-dependent cell survival by the tumor suppressor PTEN. *Cell* **95**: 29–39

Sternlicht MD, Lochter A, Sympson CJ, Huey B, Rougier JP, Gray JW, Pinkel D, Bissell MJ and Werb Z (1999) The stromal proteinase MMP3/stromelysin-1 promotes mammary carcinogenesis. *Cell* **98**: 137–146

Tetsu O and McCormick F (1999) Beta-catenin regulates expression of cyclin D1 in colon carcinoma cells. *Nature* **398**: 422–426

Toyota M and Issa JP (1999) CpG island methylator phenotypes in aging and cancer. *Semin Cancer Biol* **9**: 349–357

Wilson CL, Heppner KJ, Labosky PA, Hogan BL and Matrisian LM (1997) Intestinal tumorigenesis is suppressed in mice lacking the metalloproteinase matrilysin. *Proc Natl Acad Sci USA* **94**: 1402–1407

Yarden Y and Ullrich A (1988) Growth factor receptor tyrosine kinases. *Annu Rev Biochem* **57**: 443–478

Zhang X, Mar V, Zhou W, Harrington L and Robinson MO (1999) Telomere shortening and apoptosis in telomerase-inhibited human tumor cells. *Genes Dev* **13**: 2388–2399

Zhu J, Woods D, McMahon M and Bishop JM (1998) Senescence of human fibroblasts induced by oncogenic Raf. *Genes Dev* **12**: 2997–3007

Zindy F, Eischen CM, Randle DH, Kamijo T, Cleveland JL, Sherr CJ and Roussel MF (1998) Myc signaling via the ARF tumor suppressor regulates p53-dependent apoptosis and immortalization. *Genes Dev* **12**: 2424–2433

*© 2000 Cancer Research Campaign*

